# The Biology of *Pichia membranifaciens* Killer Toxins

**DOI:** 10.3390/toxins9040112

**Published:** 2017-03-23

**Authors:** Ignacio Belda, Javier Ruiz, Alejandro Alonso, Domingo Marquina, Antonio Santos

**Affiliations:** Department of Microbiology, Biology Faculty, Complutense University of Madrid, 28040 Madrid, Spain; ignaciobelda@ucm.es (I.B.); javiru02@ucm.es (J.R.); raalonso@ucm.es (A.A.); dommarq@ucm.es (D.M.)

**Keywords:** *Pichia membranifaciens*, PMKT, PMKT2, killer toxin, biochemical characteristics, mechanism of action, biocontrol, applications

## Abstract

The killer phenomenon is defined as the ability of some yeast to secrete toxins that are lethal to other sensitive yeasts and filamentous fungi. Since the discovery of strains of *Saccharomyces cerevisiae* capable of secreting killer toxins, much information has been gained regarding killer toxins and this fact has substantially contributed knowledge on fundamental aspects of cell biology and yeast genetics. The killer phenomenon has been studied in *Pichia membranifaciens* for several years, during which two toxins have been described. PMKT and PMKT2 are proteins of low molecular mass that bind to primary receptors located in the cell wall structure of sensitive yeast cells, linear (1→6)-β-d-glucans and mannoproteins for PMKT and PMKT2, respectively. Cwp2p also acts as a secondary receptor for PMKT. Killing of sensitive cells by PMKT is characterized by ionic movements across plasma membrane and an acidification of the intracellular pH triggering an activation of the High Osmolarity Glycerol (HOG) pathway. On the contrary, our investigations showed a mechanism of killing in which cells are arrested at an early S-phase by high concentrations of PMKT2. However, we concluded that induced mortality at low PMKT2 doses and also PMKT is indeed of an apoptotic nature. Killer yeasts and their toxins have found potential applications in several fields: in food and beverage production, as biocontrol agents, in yeast bio-typing, and as novel antimycotic agents. Accordingly, several applications have been found for *P. membranifaciens* killer toxins, ranging from pre- and post-harvest biocontrol of plant pathogens to applications during wine fermentation and ageing (inhibition of *Botrytis cinerea*, *Brettanomyces bruxellensis*, etc.).

## 1. Introduction

Killer yeast strains have the characteristic of secreting toxins of proteinaceous nature that are lethal to sensitive yeast cells and filamentous fungi. The killer phenomenon in yeasts was first discovered in *Saccharomyces cerevisiae* [1] but soon shown to be present in many other genera of yeast [2,3,4]. Until now, the killer phenomenon has been discovered in almost one hundred species of more than twenty genera [5]. Over 11 different killer toxins have been described, and they are produced by representatives of such genera as *Hanseniaspora*, *Pichia*, *Saccharomyces*, *Torulaspora*, *Ustilago*, *Williopsis*, etc. (Table 1) [6,7,8,9,10].

Killer yeasts are widespread in nature where they can be found in percentages that several fold exceed those found in laboratory strains, indicating the existence of a competitive advantage. Microbes have evolved to compete over the neighborhoods of their same niche. One of the mechanisms to gain advantage that have been described is shown by some killer yeast through the presence of dsRNA viruses. These mycoviruses encode killer toxins that provide benefits to the producing cells by killing sensitive yeast cells. Killer toxin production has been related in *S. cerevisiae* with the presence of two dsRNA viruses: L-A, the helper virus, and the M killer virus that encodes a killer toxin that determines its phenotype (K1, K2, K28 or Klus). Although killer toxins could imply an important competitive advantage, it has also been demonstrated that there is a fitness cost for carrying mycoviruses [11].

In addition to the cytoplasmic dsRNA viruses mentioned above, a variety of yeast species (*Kluyveromyces lactis*, *P. acaciae*, *P. inositovora*, *D. robertsiae*, *Trichosporon pullulans*, etc.) produce linear dsDNA plasmids, probably not encapsulated, that encoded killer toxins. Furthermore, chromosomally encoded killer toxins (*Williopsis saturnus*, *Pichia anomala*, *Pichia kluyveri*, *Pichia membranifaciens*, etc.) are also found widespread in nature. Most of these toxins exceed the focus of the presented review but their characteristics and potential applications can be reviewed in the tables showed (Table 1, Table 2 and Table 3).

The reasons why killer yeasts are immune to their own toxins remain to be elucidated for most killer systems. However, in particular cases it has been solved. For example, K28 immunity occurs via the ubiquitination of re-internalized mature toxin and unprocessed precursor moieties complexes that are degraded by the proteasome [12]. Furthermore, *Pichia acaciae*, *Kluyveromyces lactis* and *Debaryomyces robertsiae* cytoplasmic virus like elements encode toxic anticodon nucleases along with specific proteins that confer toxin immunity [13,14]. The potential use of killer yeasts and their toxins has been intended for various fields of application such as the alcohol fermentation industries (brewery, winery, and distillery), fermented vegetables, biological control of post-harvest diseases, yeast bio-typing, as antimycotics in the medical field and they have been used as model systems to understand eukaryotic polypeptide processing and expression of eukaryotic viruses [10,11,12,13,14,15,16,17,18,19,20,21].

Currently, around 1500 different species of yeasts are known [22] and one of the largest yeast genera is the genus *Pichia* within the context of number of species. The number of species included in *Pichia* genus has changed considerably since Hansen (1904) first described the genus *Pichia* [22,23,24,25]. *Pichia* species are widely distributed and are present in natural habitats and also as spoilage yeasts in several foods and beverages [26,27] and even, human pathogens [28,29].

Killer phenomenon has been discovered and studied in several species of *Pichia* that show killer activity against yeasts or fungi of clinical concern (Table 2). Toxins of the genus *Pichia* are either associated with cytoplasmic genetic elements, such as dsDNA virus-like elements or chromosomally encoded [30,31,32]. Gene products of both extranuclear and chromosomally encoded toxins, are diverse in their molecular mass. There has been described monomeric toxins of *Pichia* species from less than 3 kDa (*P. ohmeri* killer toxin) to trimeric toxins of more than 187 KDa (*P. acaciae* PaT) [29,33,34], although, similar to what occurs globally for killer toxins, the molecular mass is not generally high (20–50 KDa) in *Pichia* toxins.

The stability of most killer toxins has been shown to be restricted to acidic pH values and low temperatures [35,36,37,38]. In this regard, toxins of the *Pichia* genus are not different from the rest of killer toxins; to date, no *Pichia* toxins with stability to high temperature or wide pH ranges have been described.

Known killer toxins display diverse modes of actions, such as membrane-damaging agents, glucanase activity, inhibitors of β-1,3-glucansynthase, cell cycle arrest, inhibition of calcium uptake, or tRNase activity; and toxins of the *Pichia* genus exhibit most of them [39,40,41,42].

In initial works, it was found that the majority of yeasts isolated from spontaneously fermenting olive brines possessed the killer character and that strains of *Pichia membranifaciens*, the dominant species, were particularly active in the presence of salt, eventually influencing the development of some spontaneous fermentations [43,44]. The reasons for such an increased killer activity was elucidated in the study of the mechanism of action of the first *P. membranifaciens* toxin analyzed, called PMKT (produced by the strain CYC 1106). Additional studies indicated that *P. membranifaciens* CYC 1086, presented a different killer behavior, secreting PMKT2. This review is about the killer system of *P. membranifaciens*; and in particular, it focuses in the findings described during the last 20 years about PMKT and PMKT2, until now, the only toxins described for this species. Significant progress in the determination of the nature of the killer toxins, their toxic mechanism and approaches for the potential use of such toxins has only been done very recently. In this review, are concentrated the studies carried out for the characterization on PMKT and PMKT2, their primary and secondary receptors in sensitive strains, toxin mechanisms of action, and cellular responses of sensitive cells to killer toxins. Finally, the potential applications and future perspectives of killer toxins, with special focus in *P. membranifaciens* killer toxins, are described and discussed.

## 2. Isolation of *Pichia membranifaciens* Killer Strains

The frequency and distribution of yeasts in fermentations of several olive brines is well referenced [77,78], but investigations have habitually been related with negative effects [79,80,81]. Scarce relevance has been given to the potential positive roles that most significant yeast species can develop in olive brines, including interactions among different yeast species and with other microorganisms, particularly the predominant bacterial populations responsible for the lactic acid fermentation of olives. The majority of yeasts isolated from olive brines bore the killer character, and the strains of *P. membranifaciens*, the dominant species, were found to be particularly active [43,82]. In this anthropic environment, halotolerant yeast populations develop [43] at pH values around 4.5 and temperatures that have been observed to be compatible with the killer character shown by most yeast isolates of such environments. The presence of NaCl, in concentrations similar to that found in olive brines, increase the killer character and augment the killer spectra of yeasts isolated from this environment, assessing the relevance of the killer phenomenon and indicating that the killer character could be a competitive advantage in this environment [44]. Furthermore, the presence of killer yeast in olive brines is found to be very high (39%), indicating that, in these particular fermentative conditions, the killer character could be particularly relevant for competition [83].

The isolated *P. membranifaciens* strains were screened against a collection of several sensitive strains, for the presence of different *P. membranifaciens* killer phenotypes, determining the presence of, at least, two clearly different ones [20] and confirming the fact that different toxins can be produced by strains of the same yeast species. Two strains of *P. membranifaciens*, CYC 1106 and CYC 1086, were selected because of their different killer spectrum of resistance/sensitivity and their higher activity against the panel of potentially sensitive yeasts used. In conclusion, the strains of *P. membranifaciens* of our laboratory produced two different toxins that were named PMKT (secreted by *P. membranifaciens* CYC 1106) and PMKT2 (secreted by *P. membranifaciens* CYC 1086). These toxins are the main objective of the investigations recovered in the present work.

## 3. Production of PMKT and PMKT2

Having established that the secreted toxins increased in culture liquid as growth progressed and then stabilized when the yeast population reached the stationary phase (as considered for primary metabolites), the toxins of *P. membranifaciens* were purified from samples taken at the early stationary phase, where the toxins concentration was observed to be higher [84]. The production of killer toxins in complex media (which contained peptone, malt extract and yeast extract) and minimal media (containing growth factors, mineral salts and trace elements) has been compared. Although production of PMKT in complex media is higher than in minimal media, the specific activity is higher (seven-fold higher) in the latter case, according to similar observations that were reported previously [85,86] and probably due to the greater stability of the toxin in a media with high contain in peptides and proteins. However, for the purpose of PMKT2 production and purification, *P. membranifaciens* CYC 1086 is developed in complex medium because PMKT2 is purified easier [20,39].

In addition, with the aim of increasing toxin production, stabilizing agents such as polyols, detergents and protease inhibitors were added. Results indicated that the non-ionic detergents, in particular Brij-58, increased the killer activity present in the extracellular media after growth. Accordingly, the synthetic medium with Brij-58, was used for purification of PMKT [19]. Probably, non-ionic detergents stabilize the killer toxin produced or inhibit the adsorption by (1→6)-β-d-glucans. (1→6)-β-d-glucans in yeast cell walls are known to function as primary receptors of some killer toxins in sensitive cells [47,49,50,51,52,59,75,87]. Killer cells have these (1→6)-β-d-glucans, and it is easy to understand that some amount of the secreted toxins can be attached by (1→6)-β-d-glucans and are not released to the culture medium. The same can be applied to *KRE1* mutants, which show a 50% reduction in cell wall (1→6)-β-d-glucans that are superkillers due to an increased secretion of the K1 toxin of *S. cerevisiae* [88].

Production of toxins by killer yeasts with an increased degree of activity also depends on the culture conditions of pH and temperature. Briefly, the maximum killer toxin production for both toxins was achieved at acidic pH values of around 4.5 at 20 °C, accordingly to the activity and stability properties described later in this review for both toxins.

## 4. Homogeneity Purification and Biochemical Characterization of PMKT and PMKT2

The homogeneity purification of the killer toxins of *P. membranifaciens*, as well as other proteins and enzymes, depends, not only on the characteristics of the killer toxin, but also on the complexity of the protein extract from where they have to be purified. Furthermore, both the well-reported lability of secreted toxins from killer yeasts and their aggregation tendency are usual problems found during killer toxin purification, especially in the case of PMKT [19,20]. Accordingly, in spite of the similar biochemical characteristics of PMKT and PMKT2, the purification of both toxins is achieved using different approaches. PMKT is purified from *P. membranifaciens* CYC 1106 in four steps and being necessary the use of an affinity column of (1→6)-β-d-glucan-epoxy-Sepharose 6B [19] whereas PMKT2 is easily purified to homogeneity from *P. membranifaciens* CYC 1086 [20]. Biochemical characteristics of PMKT and PMKT2 have been determined.

Both toxins are observed to be monomeric non-glycosylated proteins of 18 kDa (PMKT) and 30 kDa (PMKT2) with isoelectric points of around 3.9 for PMKT and 3.7 for PMKT2. In addition, both toxins are found to be similar in their behavior at different pH and temperature values. Protein denaturation involves changes in protein structure (generally an unfolding) with the loss of activity. The stability of most killer toxins has been shown to be strongly dependent on acidic pH values (pH < 5.5–6.0) and low temperatures (<20–25 °C), and mechanical shaking is also destructive [35,36,37,38]. PMKT is stable only within a pH interval of 3.0 and 4.8 and it is rapidly inactivated at temperatures above 20 °C, in accordance with the loss of PMKT production during yeast growth in pH and temperatures above these values [19,20]. PMKT2 had physical chemical properties similar to PMKT: it was active at acidic pH values (2.5–4.8) and temperatures below 20 °C, although, it was less labile during short periods of time at higher temperatures and neutral pH values than PMKT. However, differences in these properties between killer toxins indicate that they are biochemically distinct [38,89]. In this regard, HM-1 killer toxin, produced by *W. saturnus* var. *mrakii* IFO0895 (previously named as *Hansenula mrakii*) is special in its wide range of stability to pH and temperature, as it remains active after exposure to 100 °C for 10 min and pH values comprised between 2 and 11 [59,90]. Probably, the observed internal disulfide bridges give to this toxin its higher stability [91,92]. *Tilletiopsis albenscens* killer toxin seems to be stable in a broad pH range of 3.5–8.0 [93].

## 5. Killing Mechanism of Action

### 5.1. Primary Receptors in Sensitive Yeast Cells

It is widely accepted that cell surface polysaccharides act as receptors for bacteria, viruses, and toxins [52,94]. Yeast killer toxins have different mechanisms of action but most toxins are able to kill sensitive yeasts in a two-step process that is receptor-mediated. There are two kinds of killer toxin receptors: the primary, usually located on the cell wall, and the secondary receptor, that exist on the plasma membrane of the sensitive cells. The interaction with the first receptor within the cell wall of a sensitive target cell involves a fast and energy-independent binding [20,49,75,76,95,96,97,98,99]. Cell walls are mainly composed by carbohydrates, some of them free and some linked to proteins. Overall, the main components of the yeast cell wall are (1→3)-β-d-glucans, (1→6)-β-d-glucans, mannoproteins and chitin, which is concentrated in the bud-scar region [100,101].

A diversity of cell wall receptors for killer toxins exist, evidencing that all the main cell wall components of yeasts could function as the primary receptor for a killer toxin. For example, (1→6)-β-d-glucans act as cell wall receptors for K1 and K2 killer toxins of *S. cerevisiae* [44,52], for *Hanseniaspora uvarum* killer toxin [102] and for *Pichia anomala* DBVPG3003. (1→3)-β-d-glucans are primary receptors for *Pichia anomala* NCYC434 [65], mannoproteins are receptors for KT28 of *S. cerevisiae* and also *Zygosaccharomyces bailii* killer toxin [63,96,103,104], and, finally, chitin is the cell receptor for *Kluyveromyces lactis* killer toxin [97]. In agreement with that, reported mutations in a gene/genes involved in the synthesis or architecture of the cell wall are observed to be resistant to killer toxins. *Kre*-Killer Resistant mutants have been obtained for K1 [105] and, in the same way, *mnn*-Mannoprotein mutants for K28 [106]. Mutational analyses revealed that both subunits of K1, α and β, are involved in glucan binding [107,108]. It has been indicated that the primary receptors in the cell wall facilitate the primary contact of the killer toxins with the sensitive yeasts or even mediates close contacts between the toxin and secondary receptors on the plasma membrane [96].

Using both in vivo and in vitro approaches, in previous works [75], it was confirmed the role of (1→6)-β-d-glucans as primary targets for PMKT and the utility of such interaction as an effective means for PMKT purification [19]. Finally, it was shown that the adsorption of PMKT to purified (1→6)-β-d-glucans occur within the first 2 min of contact, indicating, as previously hypothesized, that this process is an energy-independent binding and toxin-specific, since polysaccharides with the following linkages: (1→3)-β-, (1→4)-β-, (1→4)-α-, or (1→6)-α- were unable to bind PMKT.

Yeast mannans may be covalently linked to cell wall proteins, so that the term “mannoproteins” adequately reflects their status in the yeast cell wall. Mannoproteins have been described as antigenic determinants, also functioning as receptors or even as support for other receptors of different nature [109,110]. Conversely to what was found for PMKT, in a complementary study for the determination of the binding capacity of different yeast cell-wall fractions, cell wall glucan fractions do not bind PMKT2, whereas mannoproteins and mannan purified from sensitive yeasts were able to, showing the existence of a cell wall receptor of different nature for PMKT2 [20].

### 5.2. Secondary Receptors for *P. membranifaciens* Killer Toxins

It has been described that, for most toxins, a second step that implies toxin transfer to the plasma membrane and subsequent bind to the secondary receptor mediates killing. The number of studies conducted for the determination of the presence and description of secondary receptors are scarce. However, the evidences accumulated during years of investigation about the effects of killer toxins on sensitive yeast cells corroborated that, in addition to primary receptors, specific and essential secondary receptors should be involved in the killing mechanism. In the case of K1 toxin, the most studied killer toxin, the resistance of whole cells of *kre* mutants to K1 and the sensitivity of the spheroplasts of *kre* mutants and wild-type cells, indicate the existence of these secondary receptors [98,111]. K1 toxin, after its binding to cell wall (1→6)-β-d-glucans [85], interacts with Kre1p, an *O*-glycosylated GPI-anchored cell wall protein. Mutational analyses reveal that solely α subunit interacts with Kre1p. Δ*KRE1* mutants show complete resistance to K1, indicating that Kre1p acts as a receptor in the cytoplasmic membrane [21,98,99,103,112].

In addition, a few receptors for other toxins have been described. The H/KDEL receptor Erd2p mediates K28 toxin binding and uptake in yeast spheroplasts, a viral A/B toxin bearing an HDEL motif at the β-subunit. In vivo toxicity to K28 depends on the existence of Erd2p [113]. In the case of *Kluyveromyces lactis* zymocin, Ipt1p seems to be involved as a membrane receptor. *KTI6*, which is allelic to *IPT1* (coding for mannosyl-diinositolphospho-ceramide [M(IP)2C] synthase), join in a step that follows recognition of the primary receptor (chitin) of the toxin but precedes the role of the multi-subunit Elongator, involved in histone acetylation and transcription, the most probable toxin target [114].

Speaking specifically about *Pichia* toxins, only the membrane receptor for the PMKT toxin has been described. Some unidentified receptors have been hypothesized for other *Pichia* toxins, in an attempt to explain the binding capability and sensitivity of toxin-treated spheroplasts [115,116]. PMKT resembles K1 mechanism of action, using a similar strategy to reach the cytoplasmic membrane, involving a two-step interaction with the primary receptors (1→6)-β-d-glucans) and cell wall-localized secondary receptors which are also plasma membrane-associated. PMKT utilizes as the secondary membrane receptor a GPI-anchored protein of the cell wall [76]. This protein, called Cwp2p, can be purified using PMKT-affinity chromatography, indicating the existence of an in vitro interaction between both proteins. It was found, in a collection of 288 viable deletion mutants for proteins related to the cell periphery, as appeared in the CYGD (Comprehensive Yeast Genome Database), that 15 ORFs increased protoplast resistance to the toxin when deleted. This agrees with the hypothesis that Cwp2p is a secondary receptor for PMKT. Furthermore, complete yeast cells and *cwp2*Δ protoplasts are resistant and also unable to bind PMKT, demonstrating once again that Cwp2p is necessary for PMKT toxicity. In addition, it should be indicated that, in its definitive state, Cwp2p is bind to (1→6)-β-d-glucans and this is the most probable way by which PMKT interact closely with Cwp2p, the plasma membrane receptor located in the cell wall. It has been also demonstrated, using a collection of deletion mutants related to GPI (Glycosyl Phosphatidyl Inositol) anchor biosynthesis, that Cwp2p is present in both cellular locations the plasma membrane and cell wall, as usually occur for most GPI-proteins of the yeast cell wall [76,98]. The fact that, when exposed to PMKT, GPI anchoring-defective mutants are much more resistant, and so are protoplasts from wild-type strains previously incubated with phosphatidylinositol-specific phospholipase C (to remove GPI-anchored proteins), indicate that the precursor of Cwp2p, which is GPI-anchored, is involved in PMKT mechanism of action. Furthermore, carboxyfluorescein-entrapped liposomes that include purified Cwp2p are observed to release carboxyfluorescein in the presence of PMKT while liposomes without Cwp2p do not. This is consistent with the idea that the presence of another effector is not necessary in the proposed model. Cwp2p, could facilitate PMKT insertion into the plasma membrane bilayer, as has been proposed for other toxin receptors [117,118] and in accordance to the observed effects in PMKT-intoxicated cells [41]. The N-terminal amino acidic sequence of Cwp2p presents a PIR repeat and, due to the presence of multiple serine and threonine residues, the rest of Cwp2p is *O*-glycosylated and thus, probably bears several phosphodiester groups. At acidic pH values (those pH values where there is killer activity), PMKT bears positive charges and so on could be strongly bound to the negative charges present on Cwp2p [76]. Overall, data suggested that Cwp2p plays a central role in the activity of PMKT, acting as a bridge between the primary receptors and the plasma membrane and promoting cellular toxicity. Finally, after reaching the cytoplasmic membrane, PMKT exerts its toxin action by cation channel formation and disruption of transmembrane gradients [41]. Until now, it has not been possible to determine the existence of a secondary receptor for PMKT2.

### 5.3. Cellular Responses to Killer Toxins

#### 5.3.1. PMKT

Many targets to the killer toxin action in sensitive cells have been identified and, in consequence, some molecular mechanisms of killing action have been also determined. The first study for determining some aspects of the killing action of PMKT was intended to assess the importance of the addition of salt (which is common in the production of traditional fermented foods) in the expression of the killer phenotype [44]. In preliminary studies, we observed that sodium chloride in concentrations of up to 6% (wt/vol) eventually enhanced the toxicity and enlarged the killer spectra of a few of the yeasts tested. This observation raised the question of whether this higher activity was due to increased killer toxin production or increased sensitivity of the strains affected. In addition, it has been also shown that the killing action of some other killer yeasts isolated from high salinity environments are related on the presence of NaCl. For example, SMK toxin produced by *Pichia farinosa* KK1 shows its maximum killer activity in the presence of 2.0 M NaCl [70]. Furthermore, CnKT, produced by *Candida nodaensis*, presents a strong salt-dependent phenotype [45] and the same can also be observed for *Mrakia frigida* 2E00797 and *Williopsis saturnus* WC91-2 killer toxins [119,120]. However, so far, little has been studied concerning the reasons why, in these particular cases, the killing activity is dependent on NaCl.

PMKT finds its greatest similarity in K1, which share certain biochemical characteristics such as similar isoelectric point and molecular mass. In a similar way to the mode of action in which K1 acts from outside the cell after reaching the cytoplasmic membrane, PMKT exerts its lethal effect by ion channel formation and disruption of cytoplasmic membrane gradients [19,121,122,123]. PMKT binds to the cell wall (1→6)-β-d-glucans in the first 2–3 min after toxin addition, and then a lag phase of approximately one hour is necessary to observe reductions in cell viability, probably reflecting the time necessary for the development of the mechanism of action, gradual intoxication and death. The lag phase observed after cell wall binding and previously to cell death, is supposed to involve several sequential interactions and effects: interaction with secondary receptors, ion channel formation in the plasma membrane, alteration of transmembrane gradients and deenergization of cells and eventually, formation of large unspecific plasma membrane pores that permit the passage of other intracellular molecules [124]. During the first 30 min of incubation with PMKT, the pHi of toxin-treated sensitive cells and control cells is observed to be similar, thereafter, pHi is progressively acidified, reaching values of about 4.6–4.7 after 0.5–1 h incubation with PMKT. Then a leakage of K^+^ out of the cells starts (1–1.5 h after exposure to PMKT) and simultaneously, a flow of Na^+^ began. In the same way as K^+^, the intake of Na^+^ is directly related with killing [41]. The disruption of ionic equilibrium across the cytoplasmic membrane may could generate an augmented mortality of the cells in the presence of salt. The increased permeability of the cytoplasmic membrane to protons could consequently generate the influx of ions through the plasma membrane. Any compound accumulated in or outside the cell against its concentration gradient will equilibrate and a flow could occur. These findings are also in agreement with those studies that observed in yeast spheroplasts and artificial liposomes treated with K1 that the ion channels caused by K1 do not distinguish between Na^+^ and K^+^ [122,125].

In addition, PMKT causes in liposomes a conductance when added at low pH, showing a characteristic channel activity. Furthermore, all the common physiological ions are observed to flow through the pores caused by PMKT in the selectivity sequence: K^+^ > Na^+^ > Li^+^ > Ca^2+^ > Cl^−^. Finally, changes in plasma membrane integrity (determined as permeability to propidium iodide) are observed when cellular death was advanced in time, being probably a late consequence of PMKT treatment [41].

Yeasts response to harsh physical chemical factors (oxidative stress, heat, osmotic shock, nutrient availability, extreme pH, toxins, heavy metals, etc.) by activating a wide variety of defenses that include general or specific regulatory pathways, activating multiple MAPK cascades, which transform stimuli into appropriate physiological responses [126,127,128]. In order to confirm the physiological events described for PMKT toxicity [41], the transcriptional response of the sensitive cells was studied with a view to obtain information of the mechanisms of killing action. In addition, the results were confirmed by studying the response of deleted mutants in all those genes that were observed to be significantly up- or down-regulated when sensitive cells were treated with PMKT [73]. When the global gene expression response of the yeast *S. cerevisiae* to PMKT was explored, 146 genes showed an altered expression in response to PMKT.

**Figure 1:**
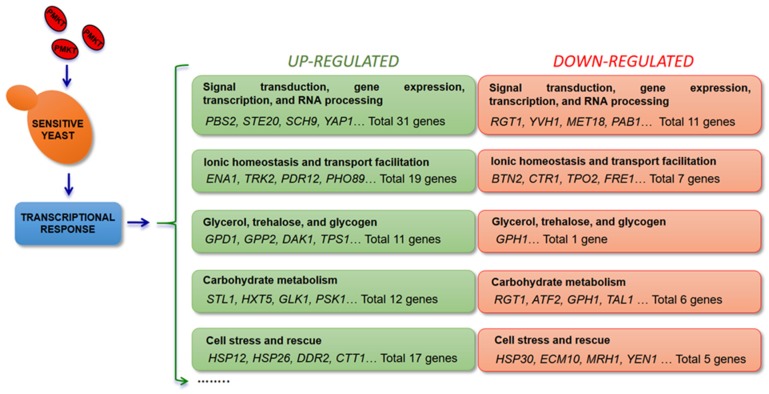
Analysis of the transcriptional response of *S. cerevisiae* to PMKT. Genes, sorted into functional categories, induced after a PMKT exposure by more than 3-fold and repressed by more than two-fold.

PMKT-treated cells with a PMKT dosage of 1205 AU/mL (considered to be a high dose as stated below) and untreated cells were selected for mRNA isolation and determination of the transcriptional response. The most important functional group with 31 up-regulated genes is the group related to signal transduction, gene expression, transcription and RNA processing, followed by the group of genes related to ionic homeostasis, transport facilitation, carbohydrate metabolism, and cell stress and rescue. Both, the phosphatases and dehydrogenases implicated in the glycerol production pathway are also up-regulated in response to PMKT. In addition, many similarities are found when the group of up-regulated genes in response to the toxin [73] and those induced by osmotic stress [129] are compared. In fact, most PMKT-induced genes encode proteins involved in counteracting the physiological changes induced by an osmotic shock, thus indicating the existence of an osmotic-related stimulus inside the cells when treated with the toxin. Genes that were related to salt tolerance in response to PMKT were *CTT1*, *HSP12*, *GPD1*, *GPP2*, *TRK2*, *PDR12*, *ENA1*, *SCH9*, *HAL9*, *YAP1*, *XBP1*, and *STL1* (Figure 1). Furthermore, *ENA1*, *TRK2*, *PHO89*, *ZRT1* and *PDR12* are a group of genes that surely are found to be up-regulated because of the leakage of ions and small metabolites induced by the toxin due to the generation of non-specific channels in a cellular response to compensate the leakage over a short period of time [41,73].

In *S. cerevisiae*, it is known that the response to rapid changes in the osmolarity relies in the activation of the HOG (High Osmolarity Glycerol) pathway, which is manifested by the phosphorylation of Hog1p. Hog1p encodes for the MAPK that is activated by phosphorylation and then translocated to the nucleus to counteract with several environmental stimuli [130]. *HOG1*Δ mutants are hypersensitive to such environmental stresses, which cause Hog1p activation [130,131]. Several transcription factors have been found to be among the substrates of Hog1p [132], so the phosphorylation and the consequent activation of Hog1p also regulates gene expression profiles [133]. Hog1p phosphorylation has been studied in order to determine whether the observed transcriptional response in the presence of PMKT was connected with the HOG pathway or not [73]. Hog1p phosphorylation is observed rapidly in PMKT-intoxicated cells. Phosphorylation is present in the first ten minutes, and during the rest of the exposure to PMKT, indicating constant activation of the signaling pathway. Accordingly, *HOG1*Δ mutants are found to be hypersensitive to PMKT.

**Figure 2:**
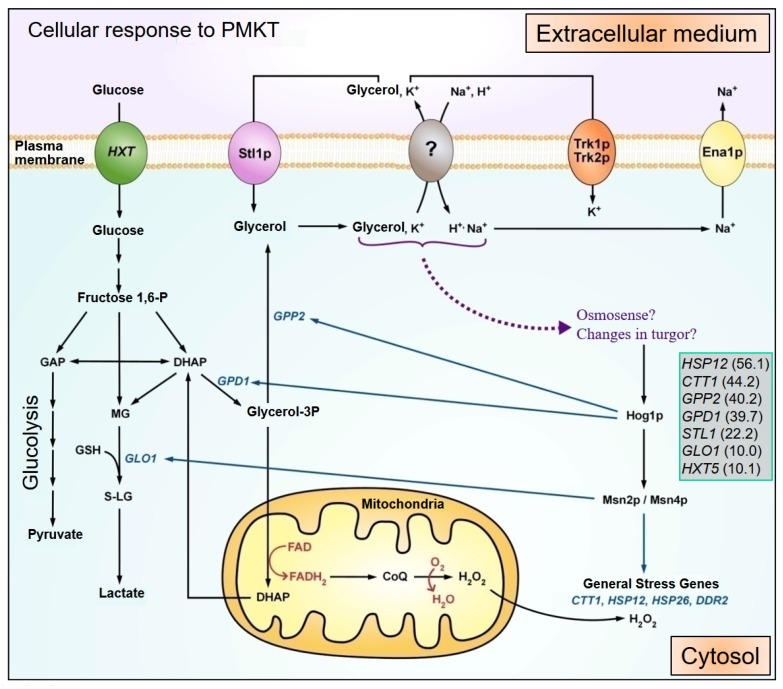
Cellular response to PMKT exposure deduced from the analysis of the transcriptional response of *S. cerevisiae* to the toxin. Once PMKT has reached the cell wall through an interaction with (1→6)-β-d-glucans, PMKT interacts with the cytoplasmic membrane through the interaction with cwp2p (not shown in the figure). This interaction, through a mechanism of unknown nature (?), leads to disruption of electrochemical gradients. Cellular receptors are not included in the scheme for simplification. PMKT cause the formation of unregulated channels through which cations and small metabolites (e.g., K^+^, H^+^, Na^+^, glycerol) flow. These changes in pH_i_, turgor or ionic concentrations generates a transcriptional response through the activation of Hog1p. Genes (*CTT1*, *HSP12*, *HSP26*, and *DDR2*), related with a general stress response, and genes (*GPD1* and *GPP2*), involved in glycerol synthesis, are transcribed to compensate the toxic effects of PMKT. Cations and glycerol, among others, can leak out from sensitive cells generating cell death. Highlighted in a box are the induction levels of the most up-regulated genes. Abbreviations: DHAP (dihydroxyacetone phosphate), G3P (glycerol-3-phosphate), FBP (fructose-1,6-bisphosphate), MG (methylglyoxal), CoQ (coenzyme Q), S-LG (S-d-lactoylglutathione), GSH (glutathione).

Glycerol is the main compatible osmolyte that accumulate rapidly as a consequence of activation of Hog1p and the transcriptional response is correlated with known responses to osmotic shocks. The mRNA coding for enzymes, *GPD1* and *GPP2*, involved in glycerol production, were augmented in the presence of PMKT, agreeing with the glycerol increment observed in response to PMKT, clearly revealing compatible osmolyte accumulation to counteract the toxin (Figure 2).

In conclusion, the most important number of up-regulated genes are core environmental stress response genes, in relation with the HOG pathway, showing that the global transcriptional response to PMKT is probably due to an alteration of the transmembrane gradients and so on cellular homeostasis [41]. Deletion mutants of up-regulated genes in response to PMKT are also observed to show hypersensitive phenotypes to PMKT, direct evidence that they are surely involved in the mechanism of action of PMKT [73], an effect also observed in HOG signaling-defective cells of *S. cerevisiae* when exposed to K1 [134], suggesting a response coincident to other hyperosmotic stresses being effective to counteract K1 toxicity. However, *GPD1*Δ mutants, and others defective in intracellular glycerol production, are hypersensitive to PMKT, but this is not the case for K1 and no downstream effectors of Hog1p relevant for K1 resistance could be identified [134]. It has been described that, for most toxins, a second energy-dependent step operates, involving toxin transference to the cytoplasmic membrane and subsequent interaction with the secondary membrane receptor.

#### 5.3.2. PMKT2

The fact that PMKT2 presented a different spectrum of killer activity, as indicated above, soon suggested that the molecular mechanism of action of PMKT2 might be different. In previous works [20], the binding of PMKT2 to mannoproteins, its primary receptors, was seen to occur in the first two or three minutes after PMKT2 incorporation and then, cellular viability reduced (80% after 5 h of exposure to PMKT2). Furthermore, none of the cellular events observed, when sensitive cells are intoxicated with PMKT, occurred with PMKT2. Leakage of K^+^, Na^+^ influx and reduction of pH_i_ were not observed during the initial exposure to PMKT2, indicating that the nature of the interaction is not related with the formation of unregulated cation-permeable channels. The observed increase of membrane permeability to H^+^, K^+^, Na^+^ after 4–5 h in contact with PMKT2 is considered an indirect effect, since they initiate concomitantly or after cell death, reflecting that they are not primary effects.

**Figure 3:**
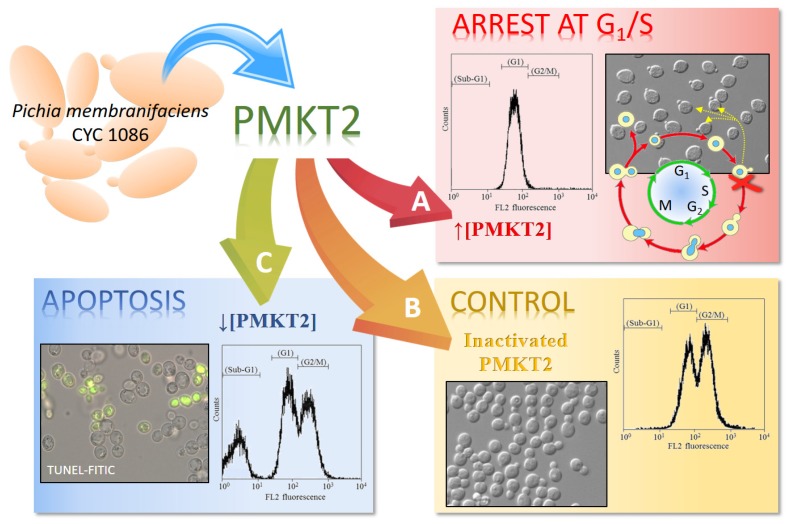
Mechanisms of the killing activity of PMKT2 depending on toxin dosage. (**A**) High concentrations of PMKT2 caused cell cycle arrest at G1/S. Flow cytometry analyses of DNA content of *S. cerevisiae* revealed that cells treated with high doses of PMKT2 were arrested at an early S phase with a nascent bud. (**B**) Asynchronously growing cells exposed to inactivated toxin remained fully viable. (**C**) Cells treated with low concentrations of PMKT2 lead to the typical markers of apoptosis. Figure adapted and reproduced with permission (Santos et al. 2013) [42].

The reason so many dead cells remain non-permeabilized during a long time of exposure to PMKT2 was elucidated when the cell cycle was studied. Flow cytometry studies of sensitive yeast cells exposed to PMKT2 show cells with arrested at an early S-phase with a nascent bud and pre-replicated 1n DNA content (Figure 3). The execution point for lethality was determined with more precision by checking the sensitivity/resistance of cells arrested at different cell cycle stages. Alpha-factor-arrested cells at the G1-phase or G2-phase-arrested cells by treatment with methyl benzimidazol-2-yl-carbamate, are resistant to PMKT2, indicating that sensitive yeasts are not affected in these stages. Furthermore, in both cases, sensitive cells rescued from the arrest are inhibited by the toxin, suggesting, all together, that G2-phase-released cells start to divide, but die in the presence of the toxin after initiating the S-phase, adopting a terminal phenotype. To explain the late ion movements across plasma membrane, we hypothesize that, during the first moments of toxin action, the cell cycle arrest does not cause cytoplasmic membrane permeabilization and then, the arrested cells became converted unviable and plasma membrane permeabilization happens [42].

In short, the killing of action of PMKT, as well as several other yeast killer toxins, is related to peptide membrane permeation properties [16,41,125], whereas the mechanism of PMKT2 is related to cell cycle arrest [42].

### 5.4. The Dual Mechanism of Action of PMKT and PMKT2

It has been described how the killing of *S. cerevisiae* sensitive cells by *P. membranifaciens* killer toxins may be expressed as a combination of two mechanisms depending on toxin dosage. High doses (between 1250 and 2000 AU/mL) of PMKT and PMKT2 are observed to induce the aforementioned effects on sensitive yeast cells. By contrast, low doses (200 AU/mL) of the toxins lead to a cell death process in *S. cerevisiae* accompanied by cytological and biochemical indicators of apoptotic cell death (PCD, Programmed Cell Death). The observed cell death process is characterized by the presence of an initial apoptotic state that rapidly evolves to a necrotic state. The rate at which this process occurs is proportional to the amount of killer toxin used in the study. Low PMKT concentrations (up to 200 AU/mL) lead to a higher number of apoptotic cells, whereas higher concentrations (from 300 to 3000 AU/mL) cause both apoptosis and necrosis. Necrotic cells are usually characterized by a permeabilized plasma membrane (usually detected by propidium iodide stain), whereas the plasma membrane that have initiated a PCD phenotype is usually intact. As a result of these investigations, it can be determined that cells appear to start the process of death with a PCD response, die as a consequence of apoptosis, and then, as a late consequence, become necrotic [74]. In these experimental conditions, cell death is characterized by cytological, genetic and biochemical indicators of apoptosis, namely, an increment of reactive oxygen species (ROS), DNA strand breaks, externalization of phosphatidylserine, metacaspase activation and cytochrome *c* release, confirming the hypothesis of PCD induction.

It has been also demonstrated that the secondary receptors in the sensitive cells have the same function in both experimental conditions. *CWP2*Δ mutants are resistant to pro-apoptotic, as well as for pro-ionophoric amounts of PMKT, also in agreement with the observations obtained for Kre1p, the plasma membrane secondary receptor involved in the K1 mechanism of action [76,135], indicating that Cwp2p is always involved in the killing action [76,135].

**Figure 4:**
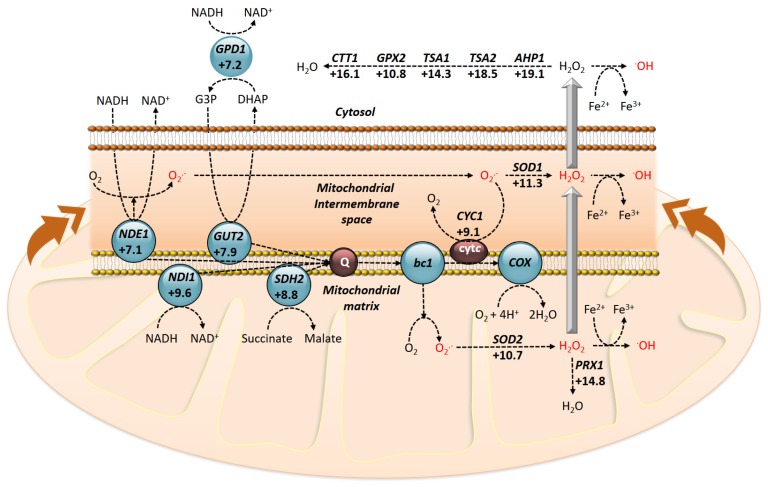
Transcriptional response of *S. cerevisiae* after exposure to pro-apoptotic concentrations of PMKT. Respiratory chain of *S. cerevisiae* with the indication of sites where ROS are formed and the main mitochondrial enzymes involved. Numbers indicate the levels of induction of some genes coding for such representative enzymes. Figure adapted and reproduced with permission (Santos et al. 2011) [74].

In the comparison studies between the two experimental conditions, it has been determined whether the transcriptional response of the cells is coincident. Therefore, using DNA microarrays, global gene expression has been also evaluated in the presence of pro-apoptotic concentrations of PMKT. The results obtained from DNA microarrays indicate that genes related with an oxidative stress response are induced, showing that the transcriptional response is not coincident with that obtained for pro-ionophoric doses of PMKT. The most highly induced genes are, in order, *DCS2*, *FLR1*, *GSH1*, *APH1*, *VPS13*, *TSA2*, *GLR1*, *ECM4*, *AAD14* and *GTO3*, most of which are observed to be induced by different oxidative compounds. Several genes encoding for ROS detoxifying enzymes, such as *CTT1*, *PRX1*, *TSA1*, and *SOD1*, are also observed to be up-regulated (activations comprised between +16.1 and +11.3), indicating an oxidative stress that can be observed by analyzing the ROS production with dihydroethidium in the presence of PMKT (Figure 4).

Taking into account the previous research on PMKT, we raised the possibility that PMKT2 also presented the aforementioned duality in its mode of action. This hypothesis was confirmed following the same experimental approach used for PMKT. Briefly, we described how the mechanism of killing of *S. cerevisiae* cells may be expressed as a sum of two mechanisms that depend on the used PMKT2 doses. High doses induce cell cycle arrest as already mentioned and low doses, as well as described for PMKT, induced a PCD process accompanied by cellular and genetic indicators of PCD in *S. cerevisiae* [42].

**Figure 5:**
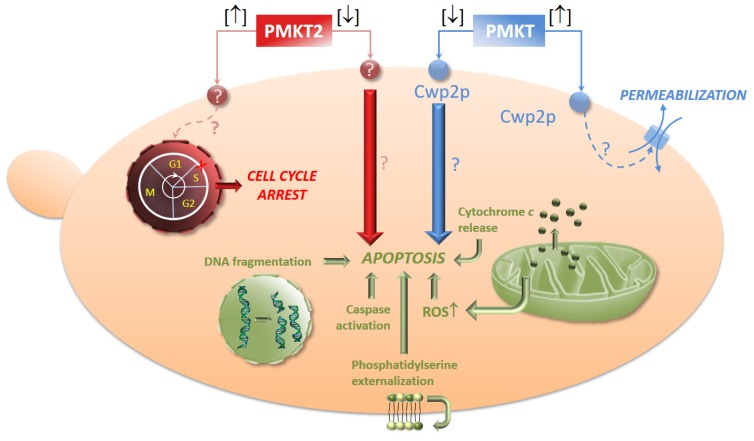
Schematic representation of the most important events known to be induced in *S. cerevisiae* after exposure to high [↑] and low [↓] concentrations of PMKT and PMKT2. Figure adapted and reproduced with permission (Santos et al. 2011; Santos et al. 2013) [42,74].

It remains to be determined why low concentrations of both toxins, PMKT and PMKT2, alter the oxidative homeostasis of sensitive cells and cause apoptosis (Figure 5). The mechanism by which PCD is induced in response to *P. membranifaciens* toxins, as other killer toxins, is as yet unknown, but it proves to be a challenging research field for the future.

## 6. Applications, Conclusions and Future Perspectives of *P. membranifaciens* Killer Toxins

Although killer toxins were originally described and studied for their ecological function in the early 1960s [1], and they promptly received much attention from geneticists for the understanding of their genetic basis at the end of this decade [86,136], their biocidal nature has an inherent applicability in clinical, food and biocontrol fields that was not systematically explored until the 1990s (for medicine: [137,138,139]; for food: [140,141,142,143]; for biocontrol: [144,145,146]). As mentioned at the beginning, *P. membranifaciens* killer yeasts exhibit a broad action spectrum including yeasts of biotechnological concern (*Brettanomyces/Dekkera*, *Candida boidinii*, *Zygosaccharomyces*) and phytopatogenic fungi (*Fusarium proliferatum*, *Botrytis cinerea*), as well as other killer yeasts [21,104].

To a large extent, the potential applications that have been proposed for yeast killer toxins have been conditioned by the aforementioned recognized lability of such toxins. The proposed applications are largely restricted to those that provide physical chemical conditions compatible with the stability of killer toxins, that is, those that can be performed at relatively low pH and low temperature values (Table 3). Although the majority of killer toxins (*W. mrakii* var. *mrakii* HM-1, *W. saturnus* toxin, *P. anomala* PaKT and *Z. bailii* zygocin) have shown activity against pathogenic yeasts, such as *Candida albicans*, *Cryptococcus neoformans*, etc., they have been suggested to be potentially useful as potential antifungal agents for infections caused by fungi, their direct application is of limited real importance because of their instability at physiological temperatures and pH values and their antigenicity [39,104,146,147,148,149,150,151]. Once again, only the killer toxins that display a remarkable stability (e.g., the *Williopsis* toxins) could be therapeutically used, but restricted to topical applications such as on superficial skin infections [151]. In this context, the role and use of *P. membranifaciens* and its killer toxins to control human or animal infections and other clinical diseases is still uncertain.

Since the use of *P. membranifaciens* killer toxins seems to be not really useful at clinical stages, their use for biocontrol purposes has a great interest in agro-food industry. The applications of the biocide activity of *P. membranifaciens* were initially unveiled by Masih et al. (2001), observing that the mycelium of the *B. cinerea* treated with *P. membranifaciens* failed to develop the characteristic grey mould symptoms when it was re-inoculated onto grapevine [152]. This destructive process was suggested to be mediated by the production of exo- and endo-β-1,3-glucanases, as was also demonstrated by Masih et al. (2002) [153]. However, as described by Santos and colleagues in both grapevine and apple matrixes, this antagonistic phenomenon could also be mediated by killer toxin (PMKT) activity [19,154]. The co-existence of different mechanisms, including hydrolytic enzymes and killer toxin production, is one of the reasons that justifies the interest and versatility of *P. membranifaciens* as a biocontrol agent against *B. cinerea*, since it was also proved effective in postharvest pears [155].

The existence of similar and additional antagonistic mechanisms in other yeasts and their use to control *B. cinerea* spoilage have also been reported. For example, the production of exo-glucanase toxins in *W. anomalus* was reported by Parafati et al. (2016) [156], but the production of certain volatile organic compounds (i.e., 2-phenylethanol) has also been suggested as determinant for the biocontrol activity against *B. cinerea* found in this species and in others such as *Candida intermedia*, *Sporidiobolus pararoseus*, *Starmerella bacilaris* and *S. cerevisiae* [157,158,159,160,161,162].

In a similar context, *P. membranifaciens* has also been used as a biological tool for the control of other crop-fruit-associated fungus pathogens such as *Penicillium expansum* in postharvest peaches (by rapid colonization and competition for space and nutrients in fruit wounds) [163] and in postharvest pears (by nutrient and space competition and the production of chitinase, glucanase and other killer activities) [155]; *Colletotrichum acutatum* causing anthracnose rot in postharvest loquat fruit (by producing chitinase and β-1,3-glucanase) [164]; *Penicillium roqueforti* in storage wheat grain (with no conclusions about the mechanism of action) [160]; and *Penicillium italicum*, *Penicillium digitatum* and *Colletotrichum gloeosporioides* causing blue and green mold and anthracnose in citrus fruit (with no conclusions about the mechanism of action) [165].

The activity of *P. membranifaciens* towards the wine spoilage yeast *B. bruxellensis* was firstly observed by Yap et al. (2000) [189], and this fact was later confirmed by Santos et al. (2009), who verified the inhibition of *P. membranifaciens* CYC 1086 and its killer toxin PMKT2 against *B. bruxellensis* in simulated winemaking conditions [20]. Apart from the use of *P. membranifaciens* CYC 1086 and PMKT2, killer toxins from *Ustilago maydis* [10], *Torulaspora delbrueckii* (TdKT) [9], *Kluyveromyces wickerhamii* (Kwkt), *Wickerhamomyces anomalus* (Pikt) [183] and *Candida pyralidae* (CpKT1) [184] and the production of pulcherriminic acid by *Metschnikowia pulcherrima* (without a real killer activity but depleting biologically available iron from the medium) [190] have been reported as useful to control *Brettanomyces* spoilage. Continuing in the enology context, results from Alonso et al. (2015) and Labbani et al. (2015) conclude that a synergistic effect was operating using PMKT [185] and Pkkp (from *P. kluyveri*) [8] killer toxins, respectively, in combination with potassium metabisulfite, against wine spoilage yeasts such as *Zygosaccharomyces* or *Dekkera/Brettanomyces* species. Recently, Velazquez and coworkers described the utility of mixed-inoculation with killer and sensitive yeast strains to speed cell death during second fermentation of sparkling-wines to release of certain compounds from autolyzed yeast cells [181].

In summary, the killer activity of *P. membranifaciens* shows a broad spectrum of potential applications, sustained by a considerable diversity of mechanisms of action. However, a marked lability of these killer toxins restricts their application to specific environments and matrixes providing adequate chemical physical conditions. These requirements are basically found in food, post-harvest, and fermentative environments, even though the industrial application of killer toxins is far from a reality, since their large-scale production is still expensive. For this reason, the current tendency is only based in applying killer yeasts instead of their toxins. Furthermore, killer resistant strains have been described since the initial studies on killer toxins and, in many surveys, it is possible to found, over a panel of potential sensitive yeast isolates, that some strains are resistant to that potential biocontrol agents [20]. In accordance, these toxins could be less valuable for biocontrol in real applications.

For those purposes, regarding toxin stability in both production and application stages, adaptive and directed evolution approaches are of great interest in the development and isolation of phenotypically improved variants. It is possible to evolve populations in laboratory conditions, where it is important that the selection conditions match the industrial parameters as closely as possible [191]. Since temperature and pH are the main limiting factors, especially for clinical applications, adaptive processes should be carried out with *P. membranifaciens* killer strains to obtain toxins with increased stability at human physiological conditions. Additionally, killer toxins identified as leads for therapeutic uses should also be modulated to reduce their antigenicity [192]. For that purpose, the molecular structure and mechanism of action of *P. membranifaciens* killer toxins should be studied in depth, in order to identify key residues causing toxicity and antigenicity, and determine their stability. Finally, the cellular response of the target pathogenic/spoilage microorganism to killer toxins needs to be studied species by species to understand the basis of their susceptibility.

Additional studies are currently under development to increase the background of knowledge accumulated during the last two decades on the features of *P. membranifaciens* killer toxins, in the hope of understanding how to work with these killer toxins that have potential biotechnological applications. Elucidation of the molecular mechanisms of their action will be helpful to develop the strategies to fight more and more harmful spoilage yeasts.

## Figures and Tables

**Table 1:** Characteristics of killer toxins from genus other than *Pichia*.

Killer Yeast	Strain (Killer Toxin)	Molecular Mass (kDa)	Glycoprotein Nature	Genetic Basis	Primary Receptors	Mode of Action	References
*Candida nodaensis*	PYCC 3198 (CnKT)	-	No	Chromosome	-	-	[45]
*Debaryomyces robertsiae*	CBS6693 (-)	>100.0		dsDNA (pWR1A pWR1B)	Chitin	-	[46]
*Hanseniaspora uvarum*	470 (-)	18.0	No	dsRNA	(1→6)-β-d-glucans	-	[47]
*Kluyveromyces lactis*	IFO1267 (Zymocin)	α (97.0); β (31.0); γ (28.0)	Yes (α), No (β, γ)	dsDNA (pGKL1)	Chitin <β2>	rRNA fragmentation	[48]
*Kluyveromyces phaffii*	DBVPG 6076 (KpKt)	33.0	Yes	Chromosome	(1→6)-β-d-glucans; (1→3)-β-d-glucans	Glucanase activity	[49]
*Saccharomyces cerevisiae*	KL88 (K1)	α (9,5); β (9.0)	No	M_1_-dsRNA	(1→6)-β-d-glucans	Ionic channels formation in plasma membrane	[50]
	- (K2)	α (21.0); β (5.0)	Yes	M_2_-dsRNA	(1→6)-β-d-glucans	Increase in plasma membrane permeability	[51,52]
	CBS8112 (K28)	α (10,5); β (11.0)	Yes	M_28_-dsRNA	Mannoproteins	Toxin entry by endocytosis and cell cycle arresting in S phase	[53]
	- (Klus)	α (?), β (?)	Yes	M_lus_-dsRNA	-	-	[54]
	111 (KHR)	20.0	-	Chromosome	-	-	[55]
	- (KHS)	75.0	-	-	-	Increase of membrane permeability to ions	[3]
*Schwanniomyces occidentalis*	ATCC 44252 (-)	α (7.4); β (4.9)	No	Chromosome	Mannoproteins	Plasma membrane damage	[36]
*Ustilago maydis*	P1 (KP1)	(19.0) α, β	-	dsRNA (P1)	-	Increase of membrane permeability to ions	[56]
	P2 (KP2)	11.1	-	-	-	Increase of membrane permeability to ions	[3]
	P4 (KP4)	13.6	-	dsRNA (P4)	-	Inhibition of Ca^+^ channels	[57]
	P6 (KP6)	α (8.6); β (9.1)	-	dsRNA (P6)	-	K^+^ depletion	[58]
*Williopsis saturnus* var. *mrakii*	IFO 0895 (HM-1)	10.7	No	Chromosome	(1→6)-β-d-glucans; (1→3)-β-d-glucans	Inhibition of b-1,3-glucan synthase	[59,60]
*Williopsis saturnus* var. *saturnus*	IFO 0117 (HYI)	9.5	No	Chromosome	-	(1→3)-b-D-glucans sintase inhibition	[61,62]
*Zygosaccharomyces bailii*	412 (KT412)	10.0	No	dsRNA	Mannoproteins	Plasma membrane damage	[63]
*Torulaspora delbrueckii*	Kbarr (Kbarr-1)	α (?), β (?), γ (?)	Yes	dsRNA	-	-	[64]
	NPCC 1033 (TdKT)	>30.0	-	-	(1→3)-β-d-glucans	Cell wall disruption and apoptotic death processes	[9]

**Table 2:** Killer toxins from genus *Pichia.*

Killer Yeast	Strain/s (Killer Toxin)	Molecular Mass (kDa)	Glycoprotein Nature	Genetic Basis	Primary Receptors	Mode of Action	Reference
*P. acaciae*	NRRLY-18665 (PaT)	α (110), β (39), γ (38)	Yes	dsDNA (pPac1-2)	Chitin	Cell cycle arrest in G1, chitinase activity	[30]
*P. anomala*	NCYC434 (Panomycocin)	49.0	Yes	?	(1→3)-β-d-glucans	(1→3)-β-d-glucan hydrolysis	[65]
	ATCC 96603/K36/UP25F (PaKT)	85.0	-	Chromosome	β-Glucan	-	[66]
	DBVPG 3003 (Pikt)	8.0	-	Chromosome	(1→6)-β-d-glucans	-	[67]
	YF07b (-)	47.0	-	Chromosome	-	(1→3)-β-d-glucanase activity	[68]
	VKM-Y (WAKTa/b)	-	-	-	-	-	[69]
*P. farinosa*	KK1 (SMKT)	α (6.6), β (7.9)	Yes	Chromosome	-	Membrane permeabilization	[70]
*P. inositovora*	NRRL Y-18709 (-)	3 subunits > 100.0	-	dsDNA (pPin1-3)	Chitin	rRNA fragmentation	[71]
*P. kluyveri*	1002 (-)	19.0	Yes	Chromosome	-	Membrane permeabilization	[37]
	DBVPG 5826 (Pkkp)	54.0	-	-	Cell wall receptor	-	[8]
*P. kudriavzevii*	RY55 (-)	39.8	-	-	-	-	[72]
*P. membranifaciens*	CYC 1106 (PMKT)	18.0	No	Chromosome	(1→6)-β-d-glucans	Membrane permeabilization/apoptosis	[19,41,73,74,75,76]
	CYC 1086 (PMKT2)	30.0	-	-	Mannoproteins	Cell cycle arrest/apoptosis	[20,38]
*Pichia ohmeri*	158 (-)	<3.0	-	Chromosome	-	Loss of cellular integrity	[33]

**Table 3:** Potential applications of killer toxins.

Applications	Organism/s	Toxin	General Description	References
**Biological models**	*Saccharomyces cerevisiae*	K28	Model for the study of proteins, lipids, and mechanisms required on endocytosis and retrograde trafficking in A/B toxins (as Ricin, Shiga, and Cholera toxins)	[166]
**Biotyping**	Killer strains panel	-	Fingerprinting and clustering yeast strains (genera *Debaryomyces*, *Kluyveromyces*, *Saccharomyces*, and *Zygosaccharomyces*) by use of killer toxin sensitive patterns	[167]
	*Saccharomyces cerevisiae*	-	Typing *S. cerevisiae* strain by combined use of RAPD and killer toxin sensitivity patterns	[168]
	*Cryptococcus laurentii* (CBS 139)	-	Biotyping varieties of *Cryptococcus neomorfans* (var. *neoformans* and var. *gattii*) by differential killer toxin sensitivity patterns	[169]
	Killer strains panel	-	Combined use of mtDNA-RFLP patterns and killer toxin biotype to study wine *S. cerevisiae* strain diversity in different viticulture regions	[170]
	Killer strains panel	-	Fingerprinting of *Saccharomyces* wine yeast by differential killer sensitivity	[171]
	*Pichia mrakii*	K9	Use *Pichia* genera killer toxins to differentiate members belonging to the *Nocardia asteroides* complex (*N. asteroides*, *N. farcinica*, and *N. nova*)	[172]
	*Pichia lynferdii*	K76		
	Killer strains panel	-	Use of toxins produced by a selected panel of killer yeast to discriminate strains belonging to genus *Candida* by their killer sensitive patterns	[173]
**Antimycotics for the treatment of human infections**	*Pichia anomala*	PaKT	Using killer toxin-like anti-idiotypic antibodies of *P. anomala* killer toxin (PaKT-antilds) to treat treating human *Pneumocystis carinii* pneumonia.	[174]
	*Zygosaccharomyces bailii*	Zygocin	Antifungal effect of Zygocin, a killer toxin produced by *Z. bailii*, on a broad-spectrum of sensitive fungal cells (*Candida*, *Pichia*, *Hanseniaspora*, *Fusarium* genera)	[104]
	*Kodamaea ohmeri* (HB55 and HB88)	-	Inhibition of *C. neoformans* (vars. *neoformans*, *grubii* and *gattii*), both clinical and environmental isolates, by two Brazilian yeasts selected for being able to inhibit human pathogenic fungi	[175]
	*Filobasidium capsuligenum*	Fc-1	Use of a novel toxin with activity against the opportunistic fungal pathogen *C. neoformans*, as a therapeutic agent for the treatment of cryptococcosis	[176]
	*Pichia anomala*	K10 MAbs	Apply of antiidiotypic monoclonal antibodies (KT MAbs) from *P. anomala* against *Aspergillus fumigatus.*	[177]
	*Williopsis mrakii* var. *mrakii* (IFO 0895)	HM-1	Activity of the killer toxin HM-1 from *W. markii* var. *mrakii* (formerly known as *Hansenula mrakii*) against *Candida* genus yeasts	[147]
	*W. saturnus* var. *mrakii* (MUCL 41968)	WmKT	Application of killer toxin (WmKT) secreted by *W. saturnus* var. *mrakii*, which is active against a wide range of pathogens.	[178]
**Antimycotics for treatment of animal infections**	*Kluyveromyces siamensis* (HN12-1)	-	Use of a killer toxin of *K. siamensis* against *Metschnikowia bicuspidata* WCY, agent for the milky disease in crab	[179]
	*Pichia anomala* (YF07b)	-	Apply of a killer toxin produced by the marine yeast *P. anomala*, against pathogenic yeast cells in crab, artemia, and shrimp	[68]
**Prevention of contaminants in fermentation industries**	*Saccharomyces cerevisiae*	-	Effect of killer strains of *S. cerevisiae* on the growth of sensitive strains during wine fermentation, to improve the selected wine strain implantation. Sparkling wines quality improvement	[180,181]
	*Kluyveromyces phaffii* (DBVPG 6076)	-	Use of *K. phaffii* killer toxin to control apiculate spoilage wine yeast (*Hanseniaspora uvarum*)	[182]
	*Torulaspora delbrueckii* (NPCC 1033)	TdKT	Application of a novel killer toxin (TdKT) from *T. delbrueckii* as a biocontrol tool in winemaking against different wine spoilage yeasts	[9]
	*Ustilago maydis* (CYC 1410)	-	Use of *U. maydis* toxins for the biocontrol of *Brettanomyces bruxellensis* for the reduction of aroma defects caused by this spoilage yeast	[10]
	*Candida nodaensis* (PYCC 3198)	CnKT	Application of CnKT in the preservation of salt-fermented foods, because of its high stability to salinity	[45]
	*D. hansenii*, *K. marxianus*, *P. anomala*, *P. guilliermondii*, *S. cerevisiae*	-	Control of olive table fermentation by the selection of killer yeast and their toxins which are able to suppress indigenous spoilage yeast growth	[86]
	*Williopsis saturnus* var. *saturnus*	-	Use of *W. saturnus* var. *saturnus* to inhibit spoilage yeast, as *S. cerevisiae* and *K. marxianus*, as a biopreservative agent on cheese making (in laboratory conditions)	[183]
	*Wickerhamomyces anomalus* (D2)	PiKT	Application of KwKT killer toxin produced by *K. wickerhamii* and PiKT produced by *W. anomalus* killer against *B. bruxellensis* during wine fermentation.	[184]
	*Kluyveromyces wickerhamii* (D15)	KwKT		
	*Candida pyralidae* (IWBT Y1140)	CpKT1	Control of *B. bruxellensis* by *C. pyralidae* killer toxin in wine fermentation.	[185]
	*Pichia membranifaciens*	PMKT	Use of PMKT to control *Zygosaccharomyces* spp. contamination in winemaking.	[186]
**Wine quality improvement**	*S. cerevisiae* (AWRI 796)	K2	Interaction of *S. cerevisiae* killer strain (AWRI 796) and sensitive strain (Tyr 303) to accelerate the autolysis in the sparkling wine production to improve the final wine quality	[187]
	*Wickerhamomyces anomalus* (CBS 1982, CBS5759)	-	Application of *W. anomalus* and *S. cerevisiae* in a mixed culture to positively influence chemical composition (higher amounts of polyphenol compounds and lower amounts of malic acid) and sensory features of produced apple wines	[188]
**Biological control**	*Kluyveromyces lactis* (PCK27)	-	Use of killer strain *K. lactis* PCK27, to inhibit *P. anomala*, aerobic spoilage yeast in silage making	[144]
	*Williopsis mrakii*	Mycocin HMK	Utilization of killer toxin HMK of *W. mrakii*, expressed in *Aspergillus niger*, to control both silage spoilage and yoghurt spoilage caused by yeasts	[90]
